# Analysis of Attention Subdomains in Obstructive Sleep Apnea Patients

**DOI:** 10.3389/fpsyt.2018.00435

**Published:** 2018-10-04

**Authors:** Eunice N. Simões, Catarina S. Padilla, Marcio S. Bezerra, Sergio L. Schmidt

**Affiliations:** ^1^Department of Neurology, Universidade Federal do Estado do Rio de Janeiro, Rio de Janeiro, Brazil; ^2^Department of Psychology, Pontifical Catholic University of Rio de Janeiro, Rio de Janeiro, Brazil; ^3^Rio Sono Clinic, Rio de Janeiro, Brazil

**Keywords:** attention deficit, obstructive sleep apnea, sleep disorders, cognitive impairments, neuropsychology

## Abstract

**Background:** Obstructive sleep apnea (OSA) is characterized by apnea–hypopnea during sleep. Overnight polysomnography (PSG) is usually used to detect the frequency of apneic and hypopneic events. Attention and executive deficits are commonly reported in OSA patients. Previous investigations suggested that cognitive impairments were dependent on attention deficits. However, attention is not a unitary domain and consists of different subdomains such as alertness, sustained attention, focused attention, and executive attention (impulsivity/hyperactivity). Little is known about the attention subdomains affected in OSA. Attention is commonly assessed using continuous performance tests, such as the continuous visual attention test (CVAT). Distinct variables can be derived from the CVAT. Each CVAT variable is associated with a specific attention subdomain.

**Objective:** This study aimed to examine the variables of the CVAT that are affected by OSA and to identify the most reliable CVAT variable that distinguishes OSA from controls via discriminant analysis.

**Method:** Patients scheduled to perform a PSG were invited to participate in this study. Immediately before the PSG, they performed the CVAT. Based on the PSG results, 27 treatment-naïve OSA patients were sampled. The same number of healthy controls were selected to match the two groups by age and gender. Five CVAT variables were examined: commission errors, omission errors, reaction time (RT), variability of reaction time (VRT), and coefficient of variability (VRT/RT).

**Results:** ANCOVAs indicated that RT and VRT were affected by OSA. No difference in accuracy (omission and commission errors) was observed between healthy controls and OSA patients. When the VRT measurements were corrected for their respective RT values (VRT/RT), the mean difference on this coefficient did not reach significance. The discriminant analysis indicated that the two groups could be best differentiated by the RT variable.

**Conclusions:** Attention problems, commonly observed in OSA patients, may reflect a primary problem on the alertness subdomain. The CVAT was able to detect the primary (alertness—RT parameter) and the secondary deficits (sustained attention—VRT parameter) associated with OSA. As there is no learning effect in the condition of retests, the CVAT can be used to assess the cognitive recovery in OSA patients during treatment.

## Introduction

Sleep disorders include diverse categories such as sleep-related breathing disorders, other respiratory disorders, sleep-related seizure disorders that do not respond to conventional therapy, narcolepsy, parasomnias, periodic limb movement during sleep, depression with insomnia, circadian rhythm disorders, and sleep-related symptoms related to neuromuscular disorders (1). In particular, obstructive sleep apnea (OSA) is characterized by reduced airflow, oxygen desaturation, episodes of cessation of breath, recurrent arousals, and impaired sleep architecture (2). Polysomnography (PSG) is routinely indicated for the diagnosis of OSA (1).

Previous studies have described a relationship between OSA and reduction in cerebral blood flow (3, 4). Abnormal cortical excitability has also been reported in OSA (5). Accordingly, cognitive impairments have extensively been described in patients with OSA (6, 7). Some investigators suggest that sleep fragmentation is responsible for cognitive impairments (8, 9) while others propose intermittent hypoxia as the determinant for the same deficits (10, 11). Thus, the effect of OSA on cognitive dysfunctions has not been fully understood (12, 13). The comprehension of this phenomenon may depend on the primary cognitive domain affected by the disorder. Tulek et al. (14) demonstrated that executive dysfunctions in OSA depend on attention deficits. Recently, this hypothesis was supported by Hvolby (15) who reported that attention deficit hyperactive disorder (ADHD) and sleep disorders share a common etiological substrate. In addition, attention impairments are commonly reported in OSA (3, 16, 17). As attention is the basic process necessary to more complex cognitive functions, it is possible that cognitive impairments in OSA patients reflect a primary deficit in attention.

Attention is the ability to choose and concentrate on relevant stimuli. Most definitions of attention are related to the selective processing of information. The biased-competition theory characterizes attention as a signal competition within the brain. Signals compete to guide behavior (18). Therefore, response accuracy and reaction time are linked to the attention construct. Accordingly, one of the most used neuropsychological tool to evaluate attention is the continuous performance test (CPT) originally developed by Rosvold et al. (19). The CPT instruments basically consist of Go/No-go tasks, and performance is measured by both accuracy (number of correct responses - number of wrong responses to No-go targets) and response times. The CPTs are highly sensitive to brain dysfunction and have been related to frontal-parietal networks (20, 21).

Mazza et al. (22) used three different vigilance tests and reported that the number of omission (failure to respond to correct targets) and commission (response to incorrect targets) errors were higher in patients with OSA. Sphirer et al. (10) using the Conners' CPT (23) showed that the performance of OSA patients was worse as compared to the normal population. Ayalon et al. (3) reported a tendency for significance in the number of commission errors using an experimental paradigm similar to the traditional CPTs. Lee et al. (24) with the aid of the psychomotor vigilance task (PVT)) found that reaction time (RT) was related to the quality of life in subjects with OSA. The PVT is a single RT performance test (25) that is commonly used in sleep research (e.g., (26)). Taken together, the results based on different performance tests remain largely controversial.

Attentional control is a complex process endowed with specific neural circuits (27). Based on these specific circuits, it has been proposed that attention consists of independent subdomains such as alertness, focused attention, impulsivity/hyperactivity (executive control), and sustained attention (27, 28). Egeland and Kovalik-Gran (28) found correlations between CPT variables and attention subdomains. More recently, Simões et al. (29) and Schmidt et al. (30) have reported that ADHD patients exhibit particular deficits on CPT variables. These authors further demonstrated that the deficits were associated with different attention subdomains. Some studies (28–30) have proposed that impaired performance on CPTs could be explained by the following four conditions: (1) a drop in vigilance caused by reduced brain activation causes slow RTs (alertness subdomain); (2) occasional lapses in attention as test progresses, affecting the stability of response times (sustained-attention subdomain); (3) failure of focused attention, severe enough to result in errors of omission (focused attention subdomain); and (4) inability to control inadequate responses (impulsivity subdomain) resulting in a high number of commission errors.

Since the CPT proved to be a valid evaluation tool, different models have been created. In this regard, the continuous visual attention test (CVAT) is a typical CPT validated for clinical use in Brazil (31). The CVAT has extensively been used in several neurological disorders such as ADHD (29, 30) and chronic pain fibromyalgia (32). It does not depend on the patient's IQ and there is no learning effect during retests. The variables derived from the CVAT include mean hit RT, variability of reaction time (VRT), omission errors (OE), and commission errors (CE) (32, 33). Huang-Pollock et al. (34) suggested that signal detection analyses of OEs and CEs are not able to address performance issues over time and stressed the use of the RT and the VRT variables. In ADHD research, VRT is typically interpreted as reflecting occasional lapses in attention (35). VRT refers to short-term changes of a person's RT on a task and can be defined as within-person inconsistency (36, 37). Since the measurement of VRT may be influenced by RT (37, 38), in the present study we analyzed the coefficient of variability (VRT/RT).

Factor analyses of different CPTs (26–31) yielded four factors that each explained a sizeable portion of variance in test scores. The four test variables that loaded highest on the four attention subdomains are the following: (1) RT loaded on the alertness factor; (2) VRT loaded on the sustained attention factor; (3) OE loaded on the focused attention factor; and (4) CE loaded on the hyperactivity/impulsivity factor. The coefficient of variability corrects any possible influence of RT on VRT.

As the variables of the CVAT assess the different subdomains of attention, this investigation aimed to examine the subdomains that were affected by OSA. We also described the variables of the CVAT that distinguished OSA patients from healthy controls via discriminant analysis. To our knowledge, this is the first study on the subdomains of attention in patients with OSA. The results would indicate if the deficits in the different subdomains of attention are independent processes or secondary to a primary deficit in a single subdomain.

## Materials and methods

### Participants

Twenty-seven OSA treatment-naïve patients of the Sleep Medicine Service/RIOSONO - RJ were selected to participate in this study. Diagnosis of OSA was established based on the PSG. OSA was defined by the apnea–hypopnea index (AHI) and the absence of other sleep disorders rather than OSA. The healthy control group consisted of subjects without sleep complaints and was sampled to match age and gender with the OSA group. Exclusion criteria for both groups were a history of psychiatric disease, alcohol or drug abuse, stroke, traumatic brain injury, pulmonary disease, epilepsy, neuromuscular diseases, and pregnancy. This study was carried out in accordance with the recommendations of the Research and Ethics committee of the Federal University of the State of Rio de Janeiro, Brazil (CAAE: 69406817.1.0000.5258). All subjects gave written informed consent in accordance with the Declaration of Helsinki.

### Polysomnography (PSG)

Sleep was monitored with a digital polysomnography (model BNT 36 / BNT Poli, manufactured by Emsamed), with the following channels: central and occipital electroencephalography (C3, C4, Cz, O1, O2, A1, and A2); submental and anterior tibial electrocardiograms (left and right tibial muscle); bilateral electrooculogram (right and left); assessment of body position, nasal airflow using a nasal cannula pressure transducer, and nasal–oral airflow using a thermistor. Respiratory effort was measured using chest and abdominal piezoelectric belts; oxyhemoglobin saturation was measured by pulse oximetry. The results of the examination were scanned and stored.

The OSA patients were classified based on the AHI. They were included in the OSA group if the AHI was <5. Respiratory events were scored blindly using the criteria of the American Academy of Sleep Medicine criteria (39): hypopneas were defined as > 50% reduction in airflow from the baseline value lasting >10 s and associated with a 3% desaturation or arousal; apneas were defined as the absence of airflow lasting >10 s. Sleep efficiency was calculated as follows: time asleep/(total time in bed–time to fall asleep).

### Attention assessment

All the participants performed a 15-min version of the CVAT version 2.0 approved for clinical use by the Brazilian Federal Council of Psychology and edited by Neurocog, Rio de Janeiro, RJ, Brazil (31). The testing equipment consisted of a laptop computer linked to a 13-inch liquid-crystal display (operating system: Windows® 10, maximum time error allowed: 30 ms) The test sessions were performed in a silent room, where only the patient and the examiner were present during the evaluation. The participants were placed in front of the computer with a distance between the center of the monitor and the eyes of approximately 50 cm. The subjects had visual acuity equal to or better than 20/30 in both eyes (glasses were used when needed). Before starting the task, the examiner instructed the subject to press the keyboard spacebar as fast as possible each time the specific stimulus (target) appears on the monitor (two geometric figures: star, target; diamond, non-target). There are 6 blocks with three subblocks, in each of 20 trials. For each block, the subblocks have different interstimulus time intervals (ISI): 1, 2, or 4 s. The order of the ISI varies between blocks. Each stimulus is displayed for 250 ms. The main goal of the CVAT is to evaluate attention performance, through the four basic quantitative variables: OE, CE, RT, and VTR. In addition, we calculated the coefficient of variability (Coef = VTR/RT).

### Study design

The subjects attending the sleep lab were invited to perform the CVAT. The patients were tested when they were admitted to the lab to perform the PSG immediately before their first night in the lab. Therefore, the experimenter was blind with respect to the final diagnosis of the participant. Then, 54 participants, 27 patients diagnosed with OSA and 27 healthy controls, were selected for the analysis of the CVAT data.

### Sample size

The formula for the sample size (Np) required to compare pairwise difference is $Np={\left[\frac{\left(Z\frac{\alpha }{2}+Z\beta \right).\sigma }{D}\right]}^{2}$, where

α: Type I error; for α = 0.05, Z$\frac{\alpha }{2}$ = 1.96;
β: Type II error; for β = 0.20, Zβ = 0.84. The β level was set at 0.20 and the power (1 – β) was 0.80;
σ: common standard deviation (based on normative data, considering age and gender);
D: minimum difference accepted, as indicated above.

For calculating the sample size, we considered the minimum differences (D). The values of these differences were estimated considering that they must reach magnitude levels that had clinical significance. Based on these assumptions and the age of the participants, we considered OEs, D = 6 errors; commission errors, D = 9 errors; reaction time, D = 100 ms; and VRT, D = 50 ms. The common standard deviation (σ) was based on previous normative data. We considered the normative data from subjects within the age range of this study and used the same gender proportion (60% females). For each of the four CVAT variables, the minimum sample size did not exceed 23 subjects.

### Statistical analysis

A multivariate analysis of covariance (MANCOVA) was used to test if OSA affected the attention performance. Dependent variables: OE, CE, RT, VRT, and Coef. Age and gender were used as covariates. Then, univariate ANCOVAS were performed using age and gender as covariates. Multiple *post hoc* comparisons were corrected using Bonferroni tests.

Comparing averages can show variable between-group test performance differences. However, they do not indicate which variables discriminate OSA from controls. Therefore, a discriminant analysis was conducted for the five CVAT variables.

Initially, the equality of the group means was tested using Wilk's λ. Then, the assumptions of the discriminant analyses were tested (linearity, normality, multilinear, equal variances, and multivariate normal distribution of the predictors). Box's M tests were performed to test the assumption of the homogeneity of covariance matrices. The test results were interpreted in conjunction with the inspection of the log determinants. Considering our sample size and the absence of outliers, we concluded that the small deviations from homogeneity groups did not violate the assumptions of the discriminant analysis.

A discriminant function (DF) was created as a linear combination of independent variables. The correlations between each variable and the DF indicated factor loadings. Then, canonical correlations were used to calculate the DF. The corresponding chi-square statistic was calculated to verify if the DF performed better than the chance level at separating the two groups (OSA vs. controls).

The standardized canonical coefficients of the discriminant function analysis were used to identify the most reliable variable for discriminating between OSA and controls. With the aid of the DF, the accuracy of the classification was measured. The sensitivity and specificity of the DF were also calculated.

Pearson correlation coefficients were calculated to provide some exploratory analyses of the relationship between the CVAT variables, AHI, and objective sleep parameters derived from the PSG.

A *p*-value of <0.05 (two-tailed) indicated significance in all studies. Analyses were performed using SPSS 21.0 for Windows (SPSS Inc., Chicago, IL).

## Results

The two groups did not differ with respect to age (*p* > 0.4) and gender (exactly the same proportion), as indicated in Table 1. Most of the patients (70%) were classified into severe or moderate OSA because only 30% of the patients (*n* = 8) exhibited a mild form of the disorder.

**Table 1:** Demography.

**Variable**	**OSA group**	**Healthy group**
Subjects	*N* = 27	*N* = 27
Age years Mean ±*SD*	49 ± 17.2	53 ± 17.9
Gender	Male (40.74%) Female (59.25%)	Male (40.74%) Female (59.25%)
AHI	Mild (8) Moderate and Severe (19)	

*OSA, obstructive sleep apnea; Age data are expressed as means ± standard deviation; The percentages of gender are presented in parentheses; AHI, apnea– hypopnea index. OSA severity was classified as mild (5 ≤ AHI ≤ 15), moderate (15 < AHI ≤ 30), or severe OSA (AHI > 30)*.

The MANCOVA showed a significant effect of the disorder (OSA vs. control) on test performance (*F* = 6.79, df = 5/46, *p* < 0.1%, η^2^ = 0.42). The gender cofactor did not reach significance (*F* = 1.13, df = 5/46, *p* = 0.36, η^2^ = 0.30). In contrast, the age cofactor was found to be significant (*F* = 3.84, df = 5/46, *p* < 1%, η^2^ = 0.11).

**Figure 1:**
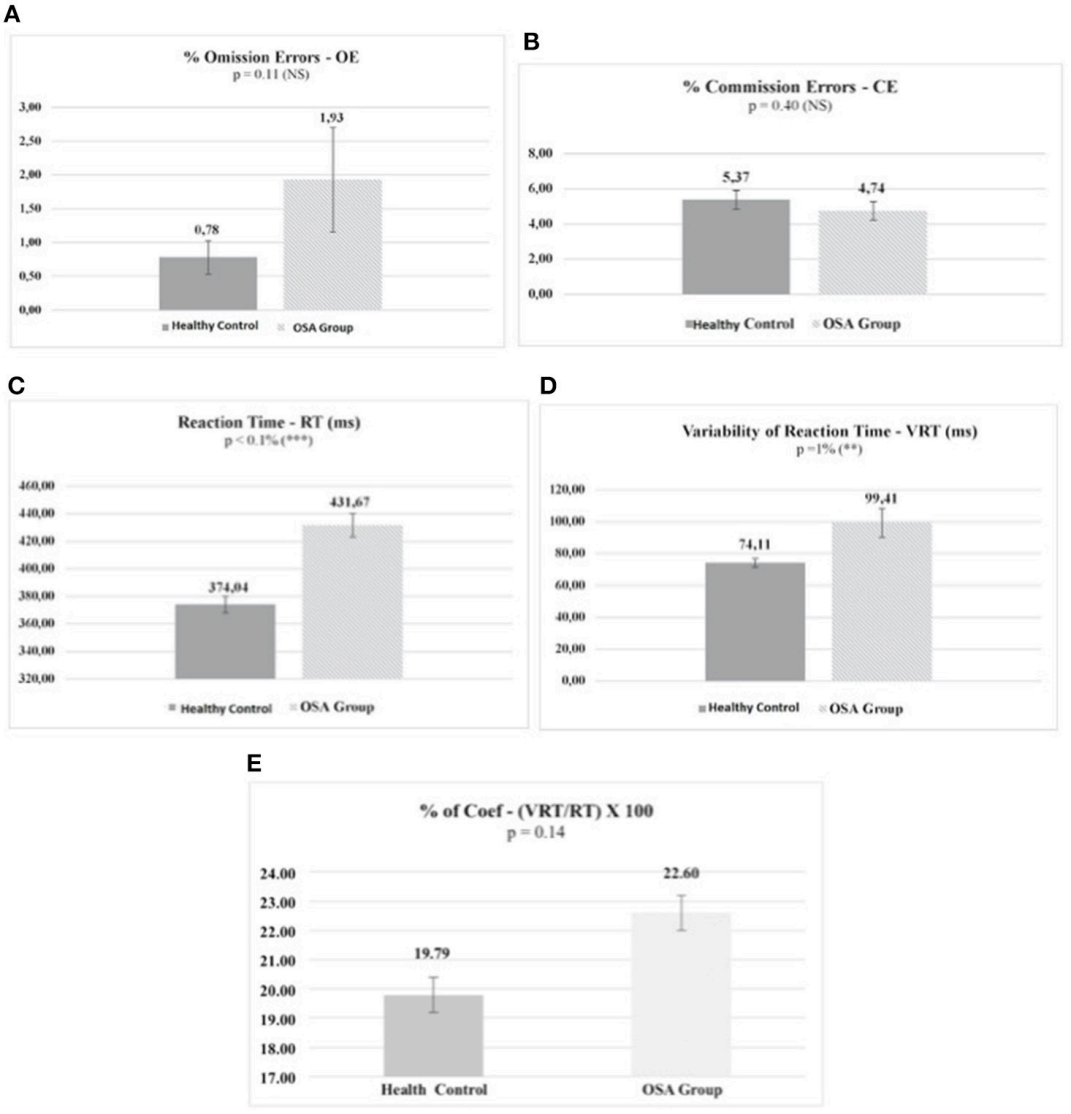
Omission errors **(A)** and commission errors **(B)** do not differ between the OSA and the healthy control groups. The OSA group exhibits a significantly higher mean RT than the control group **(C)**. The same is seem for VRT **(D)**. When the VRTs are corrected for their respective RT values (Coef = VRT/RT), the mean group difference on the quotients does not reach significance **(E)**. Values are means. Each bar represents the corresponding standard error of the mean. *P*, proof value; ms, milliseconds; NS, non-significant. Significant differences between the groups are indicated: ^**^P = 1%, ^***^P < 0.1%.

As the MANCOVA, after the correction for age and gender, reached significance for the effect of the disorder, we performed the respective univariate tests. Then, the ANCOVAs revealed that the disorder affected performance (Figure 1) for RT (*F* = 32.19, df = 1/50, *p* < 0.01%, η^2^ = 0.39) and VRT (*F* = 7.18, d = 1/50, *p* = 0.01, η^2^ = 0.13).

**Figure 2:**
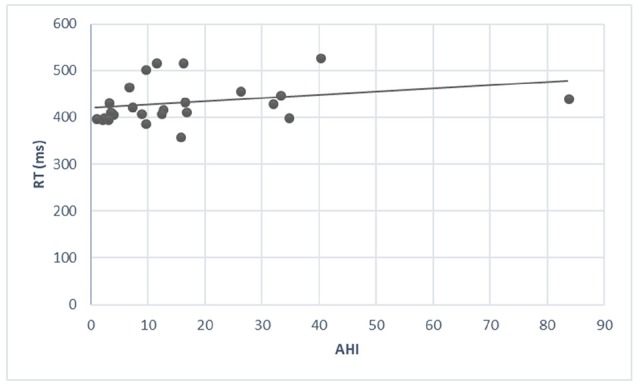
There is a modest increase in RT when AHI increases. The correlation coefficient reaches a significant level (*r* = 0.29, *P* < 5%).

There was a moderate significant correlation between AHI and RT (*r* = 0.29, *P* < 5%) (Figure 2). The correlation between AHI and the other parameters of the CVAT did not reach significant levels. An exploratory analysis of the correlations between the CVAT variables and the other objective sleep parameters derived from the PSG indicated a moderate significant correlation between sleep efficiency and OE (*r* = −0.28, *P* < 5%).

As expected, the smallest Wilk's λ was found for RT [λ _(1, 52)_ = 0.624, *P* < 0.1%], followed by VRT [λ _(1, 52)_ = 0.88, *P* = 1%]. OE, CE, and Coef did not reach significance. The structure matrix (Table 2) indicated that the loadings for OE, CE, and Coef variables did not reach the minimum value of 0.30. RT reached the highest value (0.95) followed by VRT (0.45). Among the variables considered in the discriminant equation, CE reached the least Pearson's correlation. The standardized canonical DF coefficients revealed that RT was the most reliable variable for discriminating between groups, followed by OE and VRT. The following DF was deduced from the analysis: DF = −17,71 + 0.045 ^*^ RT + 0.041 ^*^ OE−0.094 ^*^ CE−079 ^*^ VRT + 0.326 ^*^ Coef. The DF performed better than the chance level at separating OSA from healthy controls (λ = 0.64, chi square = 25.6, df = 5, *p* < 0.1%). Based on this formula, subjects with D > 0 were classified as OSA patients and those with D < 0 were classified as controls with 75.9% accuracy. Sensitivity was 81.5% and specificity 85.2%.

**Table 2:** Loadings: Pooled within-group correlations between discriminating variables and the standardized canonical discriminant function.

**Variables**	**Correlations (r)**
OE	0.24
CE	−0.14
RT	0.94
VRT	0.44
Coefficient of Variability	0.24

*(r), Pearson correlation coefficient; OE, omission errors; CE, commission errors; RT. reaction time; VRT, variability of reaction time; coefficient of variability, VRT/RT*.

## Discussion

**Figure 3:**
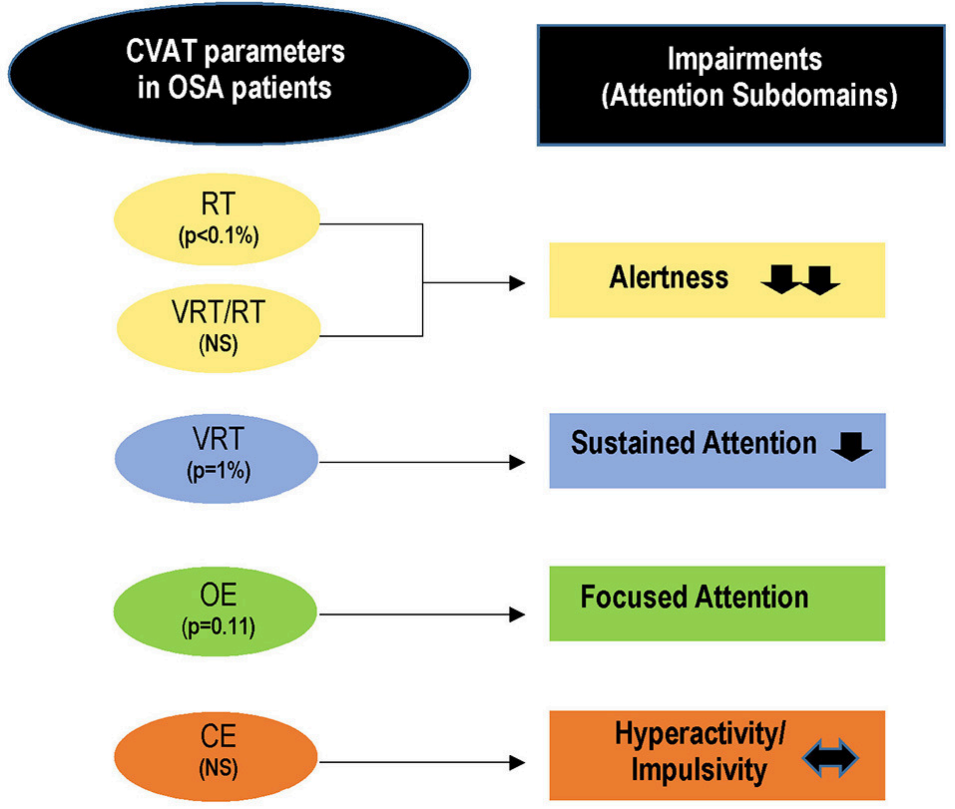
Summary of the main findings. RT and VRT are significantly higher in OSA patients as compared to the healthy control group. These data indicate impairments in alertness and sustained attention. As the ratio (VRT/RT) did not differ between the two groups, the primary impairment may be ascribed to the alertness subdomain whereas the sustained-attention impairments seem to be secondary to the decrement of alertness. OE shows a tendency to be higher in the OSA group and may suggest a secondary deficit on the focused-attention subdomain. The hyperactivity/impulsivity domain is not affected by OSA. CVAT, continuous visual attention test; OE, omission errors; CE, commission errors; RT, reaction time; VRT, variability of reaction time; P, proof value.

The ANCOVAs indicated that RT and VRT were affected by OSA. In contrast, OE and CE did not differ between health controls and OSA patients. When the VRTs were corrected for their respective RT values (VRT/RT), the results on the quotients did not reach significance. This shows that the primary variable affected by OSA is RT. In support of this result, the discriminant analysis indicated that the two groups could be better differentiated by the RT variable. Considering that the overall performance on the CVAT showed RT impairments, the data suggest a primary deficit on the alertness subdomain (Figure 3).

In contrast to the present findings, a previous study showed that RT did not differ between OSA patients and healthy controls (22). The authors used three different tests and reported that the average number of omissions and commissions were higher in OSA patients. As they did not detect significant differences in RTs, they concluded that OSA patients respond as fast as control subjects but make more mistakes. However, the three tests used by these authors are completely different from the tests used in this study. Mazza et al. (22) proposed that the absence of significant RT differences could be explained by the high difficulty level of the tests. According to these authors, the subjects made a high number of errors, which possibly mitigated RT differences. In this regard, it is well known that CEs are usually associated with faster RTs (23, 28–33). In addition, tasks with high rates of inhibition responses measure executive control rather than sustained attention (40). Therefore, the present data cannot be directly compared with those reported by Mazza et al. (22).

Ayalon et al. (3) also reported a tendency for significance in the number of CEs using a Go/No-go experimental paradigm. However, signal detection analyses of OEs and CEs in CPTs are not able to address performance issues over time (34). Wright et al. (41) analyzed performance on the basis of ability to correctly withhold response in Go/No-go tasks. They concluded that CEs in traditional CPTs are insufficiently sensitive or specific to be used individually as a diagnostic measure in various illnesses. Therefore, there is a need to consider other parameters besides OEs and CEs. Previous studies have shown that the four CPT parameters loaded on distinct attention subdomains (28–31). RT is related to the ability to respond toward impeding stimulus information (alertness subdomain). VRT is a reliable measure of the stability of information processing (37). Demonstrations of increased variability not only in the slow portion but also in the fast portion of the RT distribution suggest a potential multidimensional construct of VRT (36, 37). Taken together, these findings indicate that prolonged VRT is linked to deficits in sustained attention. A higher number of OEs were found to be related to deficits in the focused attention subdomain. CE loaded in the impulsivity subdomain. In addition, it should be mentioned that Egeland and Kovalick-Gram (28) reported that sustained attention and alertness dissociate on the Conners' CPT. Moreover, these authors did not find any significant correlation between alertness and sustained attention in several psychiatric diseases.

The process of sustaining attention is defined considering the cognitive demands associated with the continuous processing of stimuli that is related to the VRT variable. Our findings support that OSA patients naïve of treatment suffer from attention deficits because VRT was significantly affected and OE approached significant levels. Taken together, these findings support the hypothesis of a sustained attention deficit in OSA. However, VRT may be influenced by RT (37). Thus, the Coef is a convenient way to study the individual respondent's VRT controlled for RT. Wagenmakers and Brown (38) have shown that the Coef parameter controls for differences in the baseline RT. The present study showed an absence of significant difference on the coefficients between controls and OSA patients. This gives support for the hypothesis that the VRT results are explained by the RT variable. As VRT is related to lapses in attention (42, 43), we concluded that the OSA patients exhibited attention problems secondary to a primary deficit associated with RT. As this variable is associated with the alertness subdomain, we concluded that the alertness subdomain is mostly affected in OSA patients.

The RT variable would be linked predominantly to the mechanisms of the brainstem arousal systems and the reticular system to maintain alertness. These excitation systems are responsible for maintaining vigilance and good performance during the task. It has been suggested that impairments in vigilance and sustained attention during attention tasks are related to a higher rate of mental fatigue (44). In addition, several studies about mental fatigue have shown that fatigue causes negative effects on general attentional performance (45). Therefore, the greater VRT exhibited by OSA patients may reflect a fatigue state. Accordingly, Faber et al. (44) described that normal subjects performing a continuous task for more than 2 h exhibited a significant increase in their RT. In the present study, the fatigue may be caused by the sleep disorder that requires more cognitive resources for the patients to keep focused during the 15 min of the CVAT. In addition, OSA would be related to damage in brain circuits that are necessary for alertness.

The present data are in accordance with a previous study by Ayalon et al. (3). These authors have reported that a longer mean RT is associated with a higher arousal index. More recently, Lee et al. (24) have shown that the RT variable is significantly correlated with the quality of life in OSA patients. However, studies with the PVT test (25) cannot be directly compared with those derived from the CVAT. The PVT is based on the measurements of simple RTs to stimuli that occur at random intervals (10-min duration with random interstimulus intervals between 2 and 10 s). Although the PVT allows the calculation of errors of omission (i.e., lapses defined as RTs ≥ 500 ms) and errors of commission (responses without a stimulus, defined as RTs < 100 ms), it cannot be considered a typical Go/No-go test. As attention is defined as signal competition, the PVT is considered a simple RT test because it does not include the No-go stimulus.

The finding of a moderate significant correlation between AHI and RT may reflect that AHI can vary considerably from night to night. Indeed, changes in alcohol or sodium consumption or in the volume of fluid that redistributes overnight from the legs to the neck may promote the narrowing or collapse of the upper airway (46). Therefore, one would not expect a perfect correlation between AHI and RT during only one night.

This study has some limitations. First, the sample sizes were not large enough to ensure adequate power to study the influence of other cofactors on CVAT performance such as obesity and hypertension. Another limitation was the linearity Gaussian distribution assumption on RT and VRT measurements. In this regard, other investigators (47) have shown that RT distributions show sometimes prolonged RTs and consequently significant rightward skews or tails.

Another limitation might be related to the lack of comparisons of CVAT performance measurements against scores based on subjective sleepiness scales. We did not use the data derived from the subjective sleepiness scales for three reasons. First, the use of other variables derived from these scales would demand an increase in the sample size to maintain the same statistical power. Second, Frey et al. (48) reported a dissociation between subjective sleepiness and performance during sleep deprivation. Third, recent studies have stressed the limitations of OSA subjective screening instruments when making decisions about a referral for PSG (49). The present study focused on possible attention deficits caused by putative brain damage associated with OSA because this disorder is related to a significantly increased risk of a transient ischemic attack (49, 50).

In conclusion, the present findings suggest that focused attention problems, commonly observed in OSA patients, may reflect primarily a problem in the alertness subdomain. As the CVAT was able to detect the primary (alertness) and the secondary deficits (focused attention) associated with OSA, it increases the possibility to use CPTs to monitor these patients. We propose to use CPTs to follow the efficiency of therapeutic procedures to treat the disorder. This is supported by the absence of learning effects in most CPTs, including the CVAT. An adequate assessment of the different attention subdomains of attention affected by OSA would allow a better comprehension of the possible secondary cognitive consequences of this disorder. Preliminary data from our laboratory do not support an improvement in attention performance after the first night, when the patients used a continuous positive airway pressure device. However, further investigation after a longer use of the device is needed in the future.

## Author contributions

All authors listed have made a substantial, direct and intellectual contribution to the work and approved it for publication.

### Conflict of interest statement

The authors declare that the research was conducted in the absence of any commercial or financial relationships that could be construed as a potential conflict of interest.
